# Spinal Tuberculosis and Cold Abscess without Known Primary Disease: Case Report and Review of the Literature

**DOI:** 10.1155/2016/1780153

**Published:** 2016-12-13

**Authors:** Rima Patel, Vedavyas Gannamani, Emily Shay, David Alcid

**Affiliations:** ^1^Department of Medicine, Rutgers Robert Wood Johnson Medical School, 675 Hoes Lane, Piscataway, NJ 08854, USA; ^2^Department of Medicine, Saint Peter's University Hospital, 254 Easton Ave, New Brunswick, NJ 08901, USA

## Abstract

Extrapulmonary tuberculosis (TB) is uncommon but not rare. Bone and joint involvement constitute about 10% of extrapulmonary TB cases, with the spine being the most frequently affected site. Spinal TB patients typically present with back pain but other constitutional or pulmonary symptoms may be absent, rendering the diagnosis difficult. This case explores challenges in the diagnosis of spinal TB. We report a case of a 39-year-old woman presenting with vague back swelling for many years. Imaging revealed osteomyelitis of the spine but initial studies and cultures were negative for* Mycobacterium tuberculosis*. The diagnosis was confirmed weeks later when cultures demonstrated* Mycobacterium tuberculosis*. Considering the severe complications of untreated spinal TB including paraplegia and need for surgical intervention, high suspicion is critical in early diagnosis.

## 1. Introduction

Tuberculosis (TB) continues to be a common cause of infectious disease afflicting up to one-third of the world's population [1]. Even within the United States, 9,412 new TB cases were reported in 2014. Although the incidence of TB is decreasing, the size of this decrease has been the smallest in over a decade. Understandably, TB rates are higher among foreign-born persons, Asians, and persons residing in certain geographic areas including California, Texas, New York, and Florida [2].

Extrapulmonary manifestations of TB are less common but not rare. These can arise from the spread of infectious secretions through the gastrointestinal and respiratory tracts, contiguous spread, or lymphogenous and/or hematogenous dissemination [3]. Skeletal involvement accounts for about 10% of extrapulmonary tuberculosis, and, of these skeletal TB cases, the spine is the affected site in almost 50%. While in endemic countries spinal TB occurs more in older children or young adults, in developed countries, it is more prevalent in older adults. Overall, spinal TB is much less common in Western countries, but when it presents, it is primarily in the immigrant and HIV populations. The disease presents with a wide range of symptoms; back pain is the most frequent but symptoms may also include malaise, weight loss, night sweats, and neurologic signs, the most severe being paraplegia. Diagnosis requires a high degree of suspicion based on clinical findings which then warrant neuroimaging. Characteristic findings on imaging along with the presence of acid-fast bacilli on microscopy or culture confirm the diagnosis [4]. The diagnostic challenge of TB is that it is not initially considered especially in the setting of a vague clinical presentation. Here, we report an unusual case of a cold abscess as the only manifestation of tuberculosis.

## 2. Case

A 39-year-old Nigerian woman with history of sickle cell disease presented to our hospital with a chief complaint of increased swelling on her left flank for the past 2 years. She initially noticed the swelling “many years ago” as a 1 cm soft, painless mass. Over the years, the swelling progressively increased to the current size of approximately 10 cm on presentation. She reported no warmth, redness, or drainage of pus or fluid. Six months prior to presentation, the patient began experiencing a dull pain in the midline of her lower back, radiating laterally to the left lower quadrant. It fluctuated in severity but was aggravated with flexion of the back. She reported no recent fevers, fatigue, changes in weight, chills, sweats, hemoptysis, or dyspnea. She did not have any urinary or gastrointestinal symptoms nor did she experience weakness or sensory loss in her lower limbs. The patient immigrated to the United States from Nigeria one year ago. She has no known history of or exposure to TB but did receive the BCG vaccine as a child.

On examination, she was a well appearing young female. Vital signs included BP 118/60 mmHg, HR 66/min, temperature 98.8°F, O_2_ saturation 97% on room air, and BMI 20.3. She was noted to have icteric sclera and multiple enlarged, nontender, soft, mobile cervical lymph nodes (LNs), the largest being a 2 cm right anterior cervical LN. Inspection of her back revealed a 10 cm × 16 cm well-demarcated fluctuant area without erythema or other skin changes. The area was not tender to palpation, nor were there any areas of focal tenderness at the spine. Range of motion of her back and hips was fully intact. The remainder of the examination including cardiopulmonary, abdominal, and neurologic examination was unremarkable.

Review of her lab tests revealed hemoglobin of 94 g/L, MCV 92.5 fl, RDW 21.7%, reticulocyte count 0.08 of RBCs, leukocyte count 7.4 × 10^3^/*μ*L, and platelet count 317 × 10^3^/*μ*L. Other lab tests included blood urea nitrogen 2.86 mmol/L, serum creatinine 45 *μ*mol/L, AST 65 U/L, ALT 29 U/L, alkaline phosphatase 19 U/L, total bilirubin of 148.8 *μ*mol/L with indirect bilirubin 131.7 *μ*mol/L, LDH 525 U/L, and ESR 28 mm/hr. Rapid HIV testing was negative. Blood cultures drawn at initial evaluation remained sterile after 48 hours.

**Figure 1:**
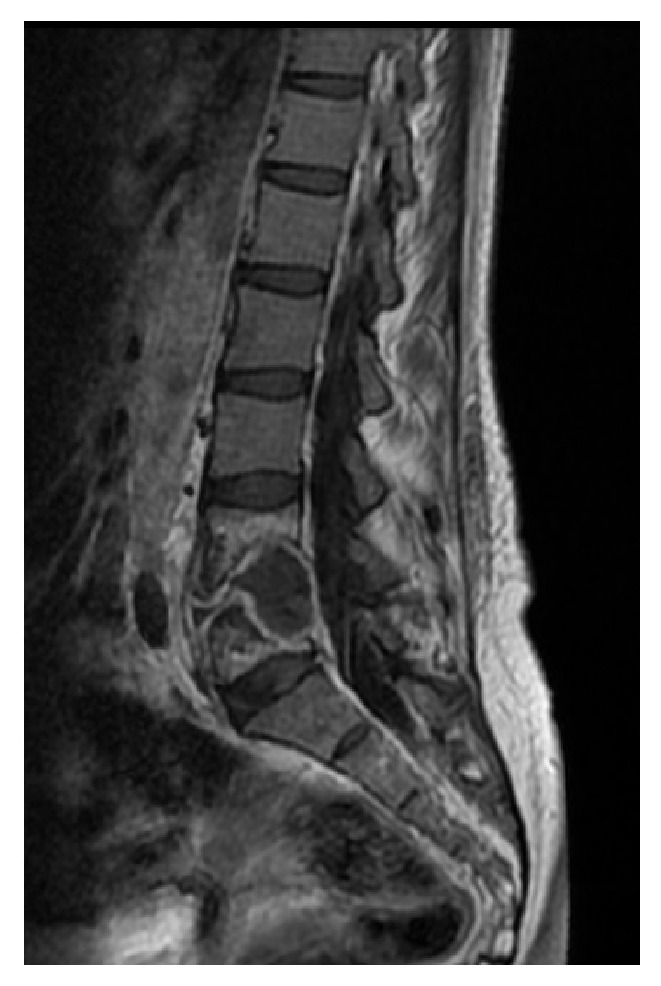
Sagittal view of MRI lumbar spine. T1 flair image with contrast demonstrating diskitis and osteomyelitis with expansile mass extending into epidural space.

**Figure 2:**
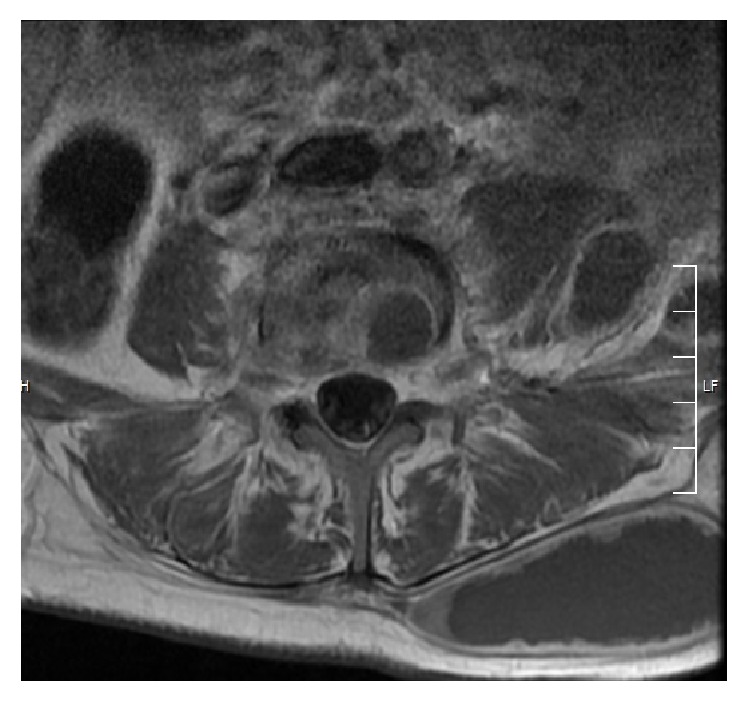
Axial view of MRI lumbar spine. T1 image with contrast depicting edema and erosive changes within vertebral body consistent with diskitis and osteomyelitis. Also figure demonstrates abscess extending into ventral epidural space.

A computed tomography (CT) scan of the abdomen and pelvis revealed diskitis/osteomyelitis at the L4-L5 level with adjacent paraspinal and left retroperitoneal abscesses extending to the left lower back/flank subcutaneous tissue and into the epidural space. A follow-up magnetic resonance imaging (MRI) of the lumbar spine with and without contrast confirmed these findings of diskitis and osteomyelitis at L4-L5 with an expansile abscess within substance of the disk extending into ventral epidural space with involvement of left L5-S1 and right L4-L5 neural foramina (Figures 1 and 2).

Ultrasound guided drainage of the abscess was attempted but only a small amount of thick tan fluid could be collected as the contents were extremely viscous. Fluid analysis showed glucose <10 mg/dL, protein 5 g/dL, and LDH 4872 U/L. Cell count was unable to be performed due to the fluid's viscosity but differential showed 82% neutrophils. Gram stain and acid-fast staining were negative for any organisms.

Given her history and presentation, a tuberculous cold abscess was high on our differential and the patient was empirically started on the standard anti-TB regimen of isoniazid, rifampin, pyrazinamide, and ethambutol. Though she had no neurological symptoms, given the extension of the abscess, both orthopedic and neurosurgery specialists were consulted for surgical drainage of abscess. The patient and neurosurgeons were initially reluctant to perform the procedure and she was therefore discharged on anti-TB medications and pyridoxine.

Upon follow-up at a local TB clinic, she complained of right upper quadrant pain and was found to have elevated transaminases. All of her medications were stopped and a week later she was restarted on rifampin and ethambutol. Four weeks from presentation, cultures from drained abscess revealed* Mycobacterium tuberculosis*. At this time, the patient was reevaluated by neurosurgery and underwent paraspinal abscess drainage through paravertebral muscles. Following the procedure, she was briefly treated with moxifloxacin as per instructions from the regional TB clinic. At her last clinic visit, six months into her treatment course, she was on isoniazid, rifampin, and pyridoxine without any complaints.

## 3. Discussion

Spinal TB is uncommon in the Western world and, hence, often overlooked by clinicians. There exist certain risk factors that should raise suspicion of TB. Agreeably, the same factors that predispose individuals to TB also increase their risk for spinal TB. These involve socioeconomic factors such as poverty, overcrowding, and illiteracy as well as conditions including malnutrition, alcoholism, diabetes mellitus, HIV, and other immunosuppressive states [4].

The most common presenting complaint of spinal TB is back pain [4, 5]. Other associated symptoms include tenderness, stiffness, muscle spasm, kyphosis from progressive bone destruction, and cold abscess, described as an abscess without the characteristic signs of inflammation. These symptoms tend to progress gradually, with average illness duration ranging from 4 to 11 months. Constitutional symptoms are not present in most cases but can include general malaise, weight loss, night sweats, and fatigue. Initial neurologic symptoms include weakness and numbness, which can progress to complete paraplegia if left untreated. The incidence of neurologic sequelae in spinal TB has been reported to vary from as low as 10% to as high as 76% [6]. The disease first affects the anterior inferior vertebral body and then progresses to involve the paradiskal, anterior, and central areas, most commonly affecting the upper lumbar and lower thoracic spines [4].

The diagnosis of spinal TB has been based upon a combination of clinical and radiological findings. MRI is considered to be the most accurate as it allows for identification of not only bone destruction but also granulomatous tissue and tuberculomas, which may be not be apparent on plain radiographs or CT. There are several imaging findings suggestive of spinal TB. Decreased signal intensity of affected bone and soft tissues on T2-weighted images with an associated thin rim enhancement of increased intensity is a pathognomonic sign for caseating necrosis or a cold abscess in TB [6]. Regardless of imaging, confirmation of the disease requires biopsy demonstrating acid-fast bacilli on microscopy or isolated culture of the organism. In contrast with pulmonary TB, extrapulmonary TB lesions have a lower amount of bacilli, resulting in less accurate results from microscopy [7]. PCR has been an effective diagnostic tool for pulmonary TB and is now thought to have high sensitivity and specificity for extrapulmonary TB as well. Compared to culture, PCR allows for a more rapid diagnosis and greater sensitivity even when small amounts of bacilli are present, as is the case with vertebral biopsies [8].

Spinal TB has a rather insidious course which often leads to greater diagnostic delay. The absence of fever, inflammatory changes, and constitutional symptoms further leads clinicians to prematurely exclude TB from their differential. Later diagnosis of the disease has been associated with a worse prognosis and a greater need for surgical intervention. Although advances in MRI should expectedly improve diagnosis time, the diagnostic delay for spinal TB has remained stable. Additionally, MRI, though valuable, does not help to differentiate between infection and malignancy [7, 9].

As with most other forms of extrapulmonary TB, antituberculous chemotherapy is the mainstay of treatment for spinal TB. However, there is no standardized regimen or known optimal duration of treatment. Therapy should initially include isoniazid, rifampin, pyrazinamide, and either ethambutol or streptomycin and can be modified based on results of susceptibility testing. Varying treatment durations ranging from 6 to 18 months have been reported [9].

The conservative approach with medical therapy is preferred for early disease, but surgical intervention may be needed to prevent neurological consequences. Neurosurgical interventions can allow for correction of deformities, abscess debridement, spinal cord decompression, or permanent spinal stabilization [10]. However, the benefits and need for surgical intervention are controversial [5]. Furthermore, there are limited guidelines to help determine management between the medical and surgical approaches [10]. A recent classification system, Gulhane Askeri Tip Akademisi (GATA), divides spinal TB into three main types (Types IA/IB, II, and III) based on seven clinical and radiological features. Type IA is the least severe with disease limited to the vertebrae and Type III the most extensive with vertebral collapse, abscess formation, and deformities. Surgery is recommended for Types IB, II, and III. Regarding cold abscesses, conservative therapy is not enough to prevent vertebral destruction; immediate drainage combined with medical therapy is needed. Epidural abscesses in particular are more likely to cause neurological issues and require urgent drainage to prevent cord compression. The classification system is considered to be a useful guide and if used during earlier stages of disease, can determine the need for surgery to prevent serious neurologic consequences such as paraplegia [11].

## 4. Conclusions

In developed countries, back pain is a frequent complaint, but it is also the most common manifestation of spinal TB. Considering the diagnosis of TB and carefully assessing risk factors can avoid delays in its diagnosis and management. This can subsequently prevent the significant neurological sequelae and need for more invasive surgical interventions.

## Abbreviations

TB: Tuberculosis
LN: Lymph node
CT: Computed tomography
MRI: Magnetic resonance imaging
LDH: Lactate dehydrogenase.
